# *Helicobacter pylori cagA* and *vacA* genes in dyspeptic Ghanaian patients

**DOI:** 10.1186/s13104-017-2542-8

**Published:** 2017-06-27

**Authors:** Timothy N. Archampong, Richard H. Asmah, Ebenezer K. Aidoo, Edwin K. Wiredu, Richard K. Gyasi, David N. Adjei, Sandra Beleza, Christopher D. Bayliss, Karen Krogfelt

**Affiliations:** 1 Department of Medicine and Therapeutics, School of Medicine and Dentistry, College of Health Sciences, University of Ghana, P O Box 4236, Korle-Bu, Accra, Ghana; 2 Korle-Bu Teaching Hospital, Accra, Ghana; 3 Department of Medical Laboratory Sciences, School of Biomedical and Allied Health Sciences, University of Ghana, Accra, Ghana; 4 Department of Medical Microbiology, School of Biomedical and Allied Health Sciences, University of Ghana, Accra, Ghana; 5 Department of Pathology, School of Biomedical and Allied Health Sciences, University of Ghana, Accra, Ghana; 6 Department of Genetics, University of Leicester, University Road, Leicester, UK; 7 Microbiology and Infection Control, Statens Serum Institut, 5 Artillerivej, Copenhagen, Denmark

**Keywords:** *Helicobacter pylori*, Endoscopy, *cagA*, *vacA*, Ghana

## Abstract

**Background:**

*Helicobacter pylori* infection is prevalent in Ghana. The development of gastro-duodenal disease is dependent on virulence of the infecting strain, host susceptibility and environmental factors. *Helicobacter pylori cagA* and *vacA* strains induce more inflammation, ulceration and oncogenesis. Here, for the first time we present data on *H. pylori cagA* and *vacA* genes and their association with gastro-duodenal disease in Ghana. A total of 159 patients with dyspepsia at Korle-Bu Teaching Hospital, Accra, were investigated for *H. pylori* with urease-CLO, of which 113 (71.1%) were positive. Genomic DNA was extracted from antral biopsies using QIAGEN DNeasy kit. Detection of *H. pylori vacA* and *cagA* genes were determined by PCR as previously described.

**Results:**

In total, 110 (69.2%) *vacA*s1, 71 (44.7%) *vacA*m1, 35 (22.0%) *vacA*m2, 77 (48.4%) *cagA*-(hydrophilic region) and 109 (68.6%) *cagA*-(internal duplication region) were detected. In multivariate analysis, duodenal ulcer was more likely than other diagnoses to have detectable *cagA*-(hydrophilic region) (OR 3.1 CI 1.2–7.9) or *vacA*s1m1 (OR 6.5 CI 1.2–34.0).

**Conclusions:**

Majority of biopsies were colonized with *H. pylori* harboring both *cagA* and *vacA*. *H. pylori cagA*-(internal duplication region) was more prevalent than *cagA*-(hydrophilic region). Duodenal ulcer was more likely than other diagnoses to have detectable *cagA*-(hydrophilic region) or *vacA*s1m1.

## Background

*Helicobacter pylori* is a spiral-shaped gram-negative urease-producing bacterium found in the gastric antrum and in areas of gastric metaplasia in the duodenum [1]. *H. pylori* infection is the cause of chronic gastritis and is important in the pathogenesis of peptic ulceration, gastric B cell lymphoma and gastric adenocarcinoma [1]. In Ghana, like many developing countries, the infection has a high prevalence rate (~80%) irrespective of the period of birth [2]. The incidence of *Helicobacter pylori* infection by rapid urease-campylobacter-like-organism (CLO)-testing in Ghanaian patients with dyspepsia referred for upper gastrointestinal endoscopy was found to be 75.4% [3].

Although infection is universally associated with gastritis, the development of clinical and endoscopic disease is dependent on a number of factors, including the virulence of the infecting strain, the susceptibility of the host and environmental co-factors [4]. The best understood bacterial virulence factor is the cytotoxin associated gene A (*cagA*) [5]. Its most hydrophilic region contains amino acid repeats including Glu-Pro-Ile-Tyr-Ala (EPIYA) [6]. In addition, the adjacent region of internal duplication has been shown to contain sequences derived from the duplication of three discrete segments of DNA [6, 7]. *Helicobacter pylori* strains possessing *cagA* induce more inflammation, ulceration and oncogenesis when compared with cag-negative strains [5]. Another such virulence factor is the vacuolating cytotoxin (*vacA*). The bacterial strains inducing more vacuolation, *vacA*s1 and m1, are closely associated with clinical disease [8].

Historically, observations postulated that gastro-duodenal disease was not common in Africa, however objective prospective endoscopic studies have demonstrated significant disease in the region [9]. Recent data also demonstrates the influence of *Helicobacter pylori* virulence factors on clinical and endoscopic disease in an endemic region like sub-Saharan Africa [10]. Here, for the first time we present data on characterization of *H. pylori cagA* and *vacA* genotypes and their association with gastro-duodenal disease in Ghanaian patients.

## Methods

The aim of the study was to utilize a cross-sectional design to characterize *H. pylori cagA* and *vacA* genes in patients with dyspepsia at the Korle-Bu Teaching Hospital, the main tertiary referral medical centre in Accra serving the majority of the southern half of Ghana. A total of 159 patients referred with dyspepsia for endoscopy at the Korle-Bu Teaching Hospital, Accra were investigated for *H. pylori* between 2012 and 2014. Patients with prior *H. pylori* eradication treatment or proton-pump inhibitor-use 2 weeks were excluded from the study. All study participants had three gastric antral biopsies taken. Biopsies were obtained from 42 patients with erosive gastritis, 38 with duodenal ulcer (DU), 16 with endoscopic suspicion of gastric adenocarcinoma (GCA), 13 with both duodenal ulcer and erosive gastritis and 50 with non-ulcer dyspepsia (NUD). Each patient had *H. pylori* infection testing on antral biopsy using rapid-urease CLO examination at upper GI endoscopy (CLO testing kit: Cambridge Life Sciences Ltd, Cambridge, UK). The additional antral samples were stored at −20 °C in DNAgard until *H. pylori vacA* and *cagA* gene analysis (Biomatrica Inc, San Diego, USA).

### *H. pylori cagA* and* vacA* genotype analysis

Genomic DNA was extracted from stored tissue samples collected from patients using QIAGEN DNeasy tissue kit, (Qiagen House, Crawley, West Sussex, UK). Detection of *vacA* and *cagA* genes was performed on gastric biopsy specimen by PCR as previously described by Rudi et al. [7].

### Statistical analysis

Means were presented for continuous variables and frequencies for categorical variables. Chi square was used to demonstrate the differences between observed variables. Bonferroni correction was made for multiple hypothesis testing with a *p*-value of <0.0017 used to indicate statistical significance. Secondary analysis was done with logistic regression to determine presence or absence of endoscopic diagnoses with specific predictor variables using SPSS 16 software.

## Results

During the study period, 159 patients with gastro-duodenal disease were screened for *H. pylori* with rapid-urease CLO examination, of which 113 (71.1%) were positive. The mean age was 49.8 years (SD 18.95). Fifty-three-percent were males and 47% females. The baseline characteristics of all patients by *vacA* and *cagA* genotype are shown in Table 1.

**Table 1:** Prevalence of *H. pylori vacA* and *cagA* genotypes in Ghanaian patients with gastro-duodenal disease

Genotype	(n)	%
*vacA*s1^+^ m^−^	44	27.7
*vacA*s1^+^m1^+^	40	25.2
*vacA*s1^+^m2^+^	13	8.2
*vacA*s1^+^m1^+^m2^+^	13	8.2
*vacAs* ^−^ m1^+^	15	9.4
*vacAs* ^−^ m2^+^	6	3.8
*vacAs* ^−^ m1^+^m2^+^	3	1.9
*cagA*13-(hydrophilic region)	77	48.4
*cagA*24-(region of internal duplication)	109	68.6
*cagA*24^+^ and *cagA*13^+^	67	42.1
*cagA* ^+^ (*cagA*13 and/or *cagA*24)	119	74.8

**Fig. 1:**
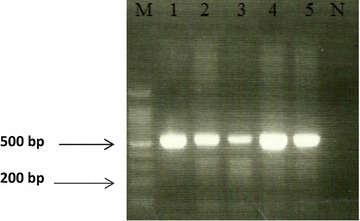
Illustrates amplicon size of 612 bp obtained for c*agA* gene-1/3 analysis. Ethidium bromide-stained 2.0% agarose gel electrophoregram of amplified *cagA* DNA fragments (612 bp) with primer cagA set 1/3. *Lane M* 100 bp molecular weight marker, *Lanes 1*–*5* PCR positives, *Lane N* negative control

In total, 110 (69.2%) *vacA*s1, 71 (44.7%) *vacA*m1, 35 (22.0%) *vacA*m2 and 119 (74.8%) *cagA* were detected. Eighty-two (74.5%) of 110 *vacA*s1 samples were *cagA*+, Fig. 1. Of the 119 *cagA*+ samples, 82 (68.9%) were found to have the *vacA*s1 genotype. The hydrophilic region and the region of internal duplication of *cagA* were detected in 77 (48.4%) and 109 (68.6%) respectively with 67 (42.1%) showing amplicons with both primer sets.

In Table 2 the relationships between *vacA, cagA* genes and endoscopic diagnoses are presented. Duodenal ulcer was associated with the hydrophilic region of *cagA* (*p* = 0.002). By contrast, non-ulcer dyspepsia was associated with a lower prevalence of *cagA*, Table 2. In multivariate analysis of all patients, duodenal ulcer was more likely than other endoscopic diagnoses to have detectable *cagA*-(hydrophilic region) (OR 3.1 CI 1.2–7.9) or *vacA*s1m1 (OR 6.5 CI 1.2–34.0), Table 3.

**Table 2:** Relationship between *vacA/cagA* genotype and endoscopic diagnoses

Genotype	Erosive gastritis		DU		GCA		NUD	
	n (%)	(*p*)	n (%)	(*p*)	n (%)	(*p*)	n (%)	(*p*)
*VacA*s1^+^	42 (75.0)	0.241	33 (64.7)	0.401	11 (68.8)	0.968	34 (69.4)	0.827
*vacA*m1^+^	25 (44.6)	0.998	24 (47.1)	0.675	6 (37.5)	0.544	21 (42.0)	0.648
*vacA*m2^+^	14 (25.0)	0.503	12 (23.5)	0.751	1 (6.2)	0.109	11 (22.0)	0.998
*vacA*s1m1^+^	19 (33.9)	0.907	20 (39.2)	0.280	5 (31.2)	0.852	13 (26.0)	0.184
*vacA*s1m2^+^	9 (16.1)	0.929	10 (19.6)	0.355	1 (6.2)	0.272	7 (14.0)	0.686
*cagA*13^+^	29 (51.8)	0.532	34 (66.7)	*0.002*	7 (43.8)	0.693	15 (30.0)	*0.001*
*cagA*24^+^	46 (82.1)	*0.007*	39 (76.5)	0.140	9 (56.2)	0.264	25 (50.0)	*0.002*

Italic values indicate significance of *p* value (*p* < 0.05)

*cagA*13-(hydrophilic region) [6, 7]

*cagA*24-(region of internal duplication) [6, 7]

**Table 3:** *H. pylori* virulence factors associated with endoscopic diagnoses in multivariate analysis

Genotype	Erosive gastritis		DU		GCA		NUD	
	OR	(CI)	OR	(CI)	(OR)	(CI)	(OR)	(CI)
*VacA*s1^+^	2.7	0.8–8.7	0.3	0.1–0.9	0.9	0.2–4.2	0.6	0.2–2.0
*vacA*m1^+^	1.9	0.5–7.5	0.3	0.1–1.1	0.4	0.04–4.5	0.5	0.2–1.8
*vacA*m2^+^	3.4	0.8–15.1	0.2	0.04–1.3	–	–	0.5	0.1–2.2
*vacA*s1m1^+^	0.4	0.1–2.1	*6.5*	*1.2–34.0*	2.2	0.2–31.4	3.8	0.8–18.8
*vacA*s1m2^+^	0.3	0.1–1.5	6.6	0.9–49.7	–	–	2.1	0.3–14.1
*cagA*13^+^	0.7	0.3–1.6	*3.1*	*1.2*–*7.9*	1.1	0.3–4.4	1.8	0.7–4.8
*cagA*24^+^	0.9	0.2–4.3	2.2	0.5–10.1	0.5	0.1–3.5	1.2	0.2–7.3

Italic values indicate Odds Ratio (OR) with Confidence Interval (CI) >1.0

*cagA*13-(hydrophilic region) [6, 7]

*cagA*24-(region of internal duplication) [6, 7]

## Discussion

In this cross-sectional study of the Ghanaian population with dyspepsia, we found a high prevalence of *H. pylori*, 71.1%, by rapid-urease CLO testing. Furthermore, the prevalence of *H. pylori* harboring the virulence factors, *cagA* and *vacAs*1, were found to be 74.8 and 69.2% respectively. The prevalence of *vacA*s1m1 was 25.2% while *vacA*s1m2 was 8.2%. Regarding the *cagA* gene, the hydrophilic region was more likely to be detected in duodenal ulcers while the region of internal duplication was associated with erosive gastritis, suggesting the effect of these genotypes in disease development. The significant association between *cagA*-(hydrophilic region) and duodenal ulcer persisted following multivariate analysis.

Majority of *vacA*s1^+^ samples were *cagA* ^+^ which was consistent with other studies [6, 11]. The expression of *cagA* gene is closely associated with that of vacuolating cytotoxin A (*vacA*) [11]. *H. pylori cagA* had a high prevalence in this study, 74.8%. This was demonstrated in other studies in Nigeria 90% [12], South Africa 95% [13]. Most *H. pylori* strains can be classified into two major groups. Type 1 have the gene coding for *cagA* and co-express *cagA* and *vacA*. Type 2 do not have the *cagA* gene and do not express *cagA* or *vacA* [14]. However, an intermediate phenotype has been identified expressing *cagA* independent of *vacA* or vice versa [14]. In Ghana the infecting *Helicobacter* pylori are seen to be of type 1.

The vacuolating cytotoxin A contains at least two variable regions, the signal (s) region which encodes the signal peptide and the middle (m) region [15]. The s region has two sub-types s1 and s2 while the m region has m1 and m2 [15]. The amount of cytotoxin produced is highest in the *vacA*s1m1 allele followed by the *vacA*s1m2 [16]. The frequency of *vacA*s1 and m1 vary across populations. The *vacA*s1 and m1 genes have been detected at a higher frequency in isolates from patients with DU in comparison with *vacA*s2 and m2, mostly in countries with a relatively low prevalence of *H. pylori* infection but also in South America and South Africa where the infection is endemic [16].

In our study population, *vacA*s1m1 genotype had a prevalence of 25.2%. Other African studies in Nigeria and Ethiopia had *vacA*s1m1 prevalence of 24 and 48% respectively [12]. Infection with *H. pylori* strains having the *vacA* s1m1 genotype (compared with s1m2 and s2m2) have also been associated with an increased risk of duodenal ulcer disease [17] as evident in this study. However, other reports from *H. pylori* endemic countries have not shown a significant association between *vacA*s1 or *vacA*s1m1 and gastro-duodenal disease [12]. A complex interplay of host genetic factors, environmental factors and other virulence factors of *H. pylori* are therefore important in determining the risk of gastro-duodenal disease [18].

*CagA* demonstrates considerable diversity in its 3′ region. Its most hydrophilic region contains amino acid repeats including Glu-Pro-Ile-Tyr-Ala (EPIYA) [6]. In addition, the adjacent region of internal duplication has been shown to contain sequences derived from the duplication of three discrete segments of DNA (D1, D2 and D3) [6, 7]. In this study, patients with duodenal ulcer were more likely to have detectable *H. pylori cagA*-(hydrophilic region) while *H. pylori cagA*-(internal duplication region) was associated with erosive gastritis. The PCR products of the region of internal duplications of *cagA*+ differ in size varying from 450 to 558 bps. *H. pylori* has been shown to express both gene regions in majority (98%) of patients [7]. However, in this study, *cagA*-(internal duplication region) was more prevalent than *cagA*-(hydrophilic region), (68.6% vs 48.4%) and 42.1% had both gene regions. This significant association between *cagA*-(hydrophilic region) and duodenal ulcer persisted following multivariate analysis.

Differences in *cagA* gene in our study population imply heterogeneity in the *cag*-pathogenicity island which may have an impact on clinical disease. This study however had limitations; PCR analysis was done on genomic DNA isolated from gastric biopsies which may have underestimated the *vacA* and *cagA* prevalence in the study population due to potential PCR inhibitors. Further evaluation would be required to characterize *H. pylori* bacterial diversity including cag-pathogenicity island gene polymorphisms and their impact on gastro-duodenal disease.

## Conclusion

Majority of biopsies were colonized with *H. pylori* harboring both *cagA* and *vacA*. *H. pylori cagA*-(internal duplication region) was more prevalent than *cagA*-(hydrophilic region). Duodenal ulcer was more likely than other diagnoses to have detectable *cagA*-(hydrophilic region) or *vacA*s1m1.

## Abbreviations

CLO: campylobacter-like organism
*CagA*: cytotoxin associated gene A
*vacA*: vacuolating cytotoxin A
DU: duodenal ulcer
GCA: gastric cancer
NUD: non-ulcer dyspepsia
